# Riluzole, a Derivative of Benzothiazole as a Potential Anti-Amoebic Agent against *Entamoeba histolytica*

**DOI:** 10.3390/ph16060896

**Published:** 2023-06-19

**Authors:** Maritza Velásquez-Torres, José Guadalupe Trujillo-Ferrara, Marycarmen Godínez-Victoria, Rosa Adriana Jarillo-Luna, Víctor Tsutsumi, Virginia Sánchez-Monroy, Araceli Posadas-Mondragón, Roberto Issac Cuevas-Hernández, José Angel Santiago-Cruz, Judith Pacheco-Yépez

**Affiliations:** 1 Sección de Estudios de Posgrado e Investigación, Escuela Superior de Medicina, Instituto Politécnico Nacional, Ciudad de Mexico 11340, Mexico; mvelasquezt1500@alumno.ipn.mx (M.V.-T.); mgodinezv@ipn.mx (M.G.-V.); vsanchezm@ipn.mx (V.S.-M.); 2 Laboratorio de Investigación en Bioquímica, Escuela Superior de Medicina, Instituto Politécnico Nacional, Ciudad de Mexico 11340, Mexico; jtrujillo@ipn.mx (J.G.T.-F.); rcuevash@ipn.mx (R.I.C.-H.); 3 Coordinación de Ciencias Morfológicas, Escuela Superior de Medicina, Instituto Politécnico Nacional, Ciudad de Mexico 11340, Mexico; rajarillo@ipn.mx; 4 Departamento de Infectómica y Patogénesis Molecular, Centro de Investigación y de Estudios Avanzados del Instituto Politécnico Nacional, Ciudad de Mexico 07360, Mexico; vtsutsu@cinvestav.mx; 5 Laboratorio de Medicina de Conservación, Sección de Estudios de Posgrado e Investigación, Escuela Superior de Medicina, Instituto Politécnico Nacional, Ciudad de Mexico 11340, Mexico; aposadasm@ipn.mx; 6 Laboratorio de Ecología Microbiana, Escuela Nacional de Ciencias Biológicas, Instituto Politécnico Nacional, Ciudad de Mexico 11350, Mexico; jsantiagoc@ipn.mx

**Keywords:** *Entamoeba histolytica*, anti-amoebic activity, riluzole, benzothiazole

## Abstract

Amoebiasis is produced by the parasite *Entamoeba histolytica*; this disease affects millions of people throughout the world who may suffer from amoebic colitis or amoebic liver abscess. Metronidazole is used to treat this protozoan, but it causes important adverse effects that limit its use. Studies have shown that riluzole has demonstrated activity against some parasites. Thus, the present study aimed, for the first time, to demonstrate the in vitro and in silico anti-amoebic activity of riluzole. In vitro, the results of *Entamoeba histolytica* trophozoites treated with IC_50_ (319.5 μM) of riluzole for 5 h showed (i) a decrease of 48.1% in amoeba viability, (ii) ultrastructural changes such as a loss of plasma membrane continuity and alterations in the nuclei followed by lysis, (iii) apoptosis-like cell death, (iv) the triggering of the production of reactive oxygen species and nitric oxide, and (v) the downregulation of amoebic antioxidant enzyme gene expression. Interestingly, docking studies have indicated that riluzole presented a higher affinity than metronidazole for the antioxidant enzymes thioredoxin, thioredoxin reductase, rubrerythrin, and peroxiredoxin of *Entamoeba histolytica*, which are considered as possible candidates of molecular targets. Our results suggest that riluzole could be an alternative treatment against *Entamoeba histolytica*. Future studies should be conducted to analyze the in vivo riluzole anti-amoebic effect on the resolution of amebic liver abscess in a susceptible model, as this will contribute to developing new therapeutic agents with anti-amoebic activity.

## 1. Introduction

Amoebiasis is an infection of the gastrointestinal tract caused by the protozoan parasite *Entamoeba histolytica* (*E. histolytica*), which can invade the large intestine and produce acute intestinal diseases, such as amoebic colitis, or more severe clinical conditions, such as amoebic dysentery. Additionally, it can spread in a hematogenous manner to other organs, such as the lungs, brain, and liver, where extraintestinal necrotic lesions, such as an amoebic liver abscess (ALA) [1], give rise to high rates of morbidity and mortality worldwide [2].

Metronidazole (MTZ) is the primary drug used for amoebiasis treatment [3]. However, treatments for prolonged periods and at high doses cause side effects in patients, such as nausea, a metallic taste, loss of appetite, diarrhea, and headache [4]. In addition, MTZ can induce other adverse effects, such as genotoxicity, teratogenicity, mutagenicity, carcinogenicity [5,6,7], and neurotoxicity [8]. Consequently, treatments may be interrupted, and the infection will not be completely eradicated [9]. In addition, some strains of amoebae have been reported to be resistant to metronidazole in vitro [10] and in vivo [11]. Considering the aforementioned, it is necessary to explore new alternative treatments from biological or synthetic sources that present high anti-amoebic activity and are innocuous for humans or for which it is possible to minimize the adverse and toxic effects.

Benzothiazoles are heterocyclic compounds constituted [12] from two heterocyclic rings containing nitrogen and sulfur. These functional groups give them the property of binding with high affinity to various receptors. Additionally, modulating drug properties such as polarity, solubility, and lipophilicity has been extensively investigated during the development of new drugs with the desired properties [13].

Among the beneficial biological and pharmacological properties of benzothiazole derivatives are their anticancer [14], antimicrobial [15], antidiabetic [16], anticonvulsant [17], anti-inflammatory [18], antiviral [19], and antitubercular activities [20]. In relation to their antiparasitic activities, they have been described as [21,22,23] leishmanicidal [24,25], anthelmintic [26], antimalarial [27], and trypanocidal [28,29,30].

Riluzole (RLZ) (2-amino 6 (trifluoromethoxy) benzothiazole) is a benzothiazole derivative, and its use has been approved by the FDA to treat amyotrophic lateral sclerosis [31]. Some pharmacological mechanisms of RLZ are (i) blocking voltage-dependent sodium channels in a dose-dependent manner; (ii) the inhibition of glycolytic enzymes; (iii) DNA damage; and (iv) the inhibition of topoisomerase II, tyrosine kinase, and the proteasome–ubiquitination system [32,33,34,35,36]. However, the amoebicidal effect of riluzole is unknown. In the present research, for the first time, an in vitro anti-amoebic activity evaluation of riluzole on *E. histolytica* trophozoites was performed, and a possible mechanism of action against amoebic molecular targets was noted.

## 2. Results

### 2.1. Riluzole Decreases the Viability of E. histolytica Trophozoites

**Figure 1:**
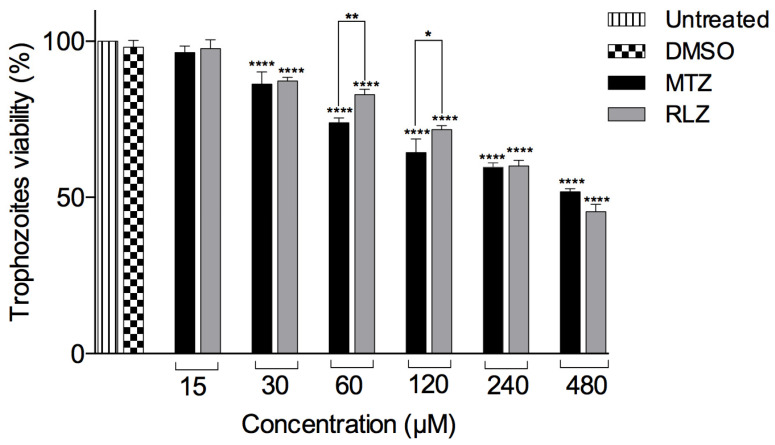
Effect of riluzole on *E. histolytica* trophozoites. Trophozoites were treated with different concentrations of riluzole (RLZ) or metronidazole (MTZ) or untreated or treated with 0.01% DMSO incubated for 5 h at 37 °C. The viability was determined by WST-1. The data are representative of two independent experiments that were performed in triplicate, and the error bars represent the mean ± SD. ANOVA analysis was used to compare the treatments, and statistical significance was defined as * *p* < 0.05, ** *p* < 0.01, and **** *p* < 0.0001.

Our results showed that RLZ decreased the amoeba viability in a dose-dependent manner, in which a concentration of 480 μM reduced cell viability by 48.1% in comparison with the control cells growing in TYI-S33 medium and DMSO (Figure 1), showing the anti-amoebic activity of RLZ. Likewise, the RLZ IC_50_ was determined to be 319.5 μM, and the MTZ IC_50_ was 459.5 μM. These concentrations were used for the following experiments presented below.

### 2.2. Riluzole Treatment Does Not Affect the VERO Cell Line

**Figure 2:**
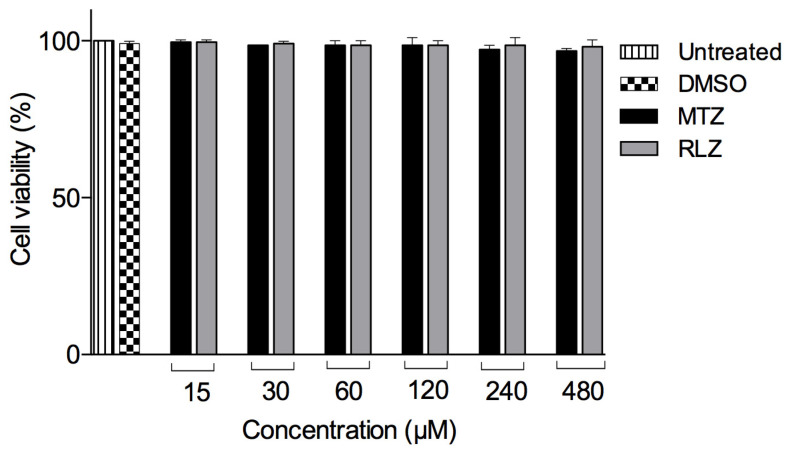
The cytotoxicity of riluzole on a VERO cell line. VERO cells were treated with different concentrations of riluzole (RLZ), and after 5 h of exposure, the WST-1 assay was performed, and the cell viability was determined. The data are representative of two independent experiments that were performed in triplicate; no statistically significant viability was observed in the VERO cell line compared to the other groups.

An in vitro cytotoxicity assay was performed on the VERO cells using the WST-1 technique to verify that this compound did not affect the viability of a cell line. There were no significant differences in the cell viability at 15, 30, 60, 120, 240, and 480 μM RLZ after 5 h in comparison with the control cells growing in D-MEM/F-12 medium and DMSO (Figure 2). These results showed that, at the tested RLZ concentrations, this compound did not decrease the VERO cell viability. Therefore, RLZ does not reduce the viability of VERO cells.

### 2.3. Riluzole Treatment Induces Ultrastructural Changes in E. histolytica Trophozoites

**Figure 3:**
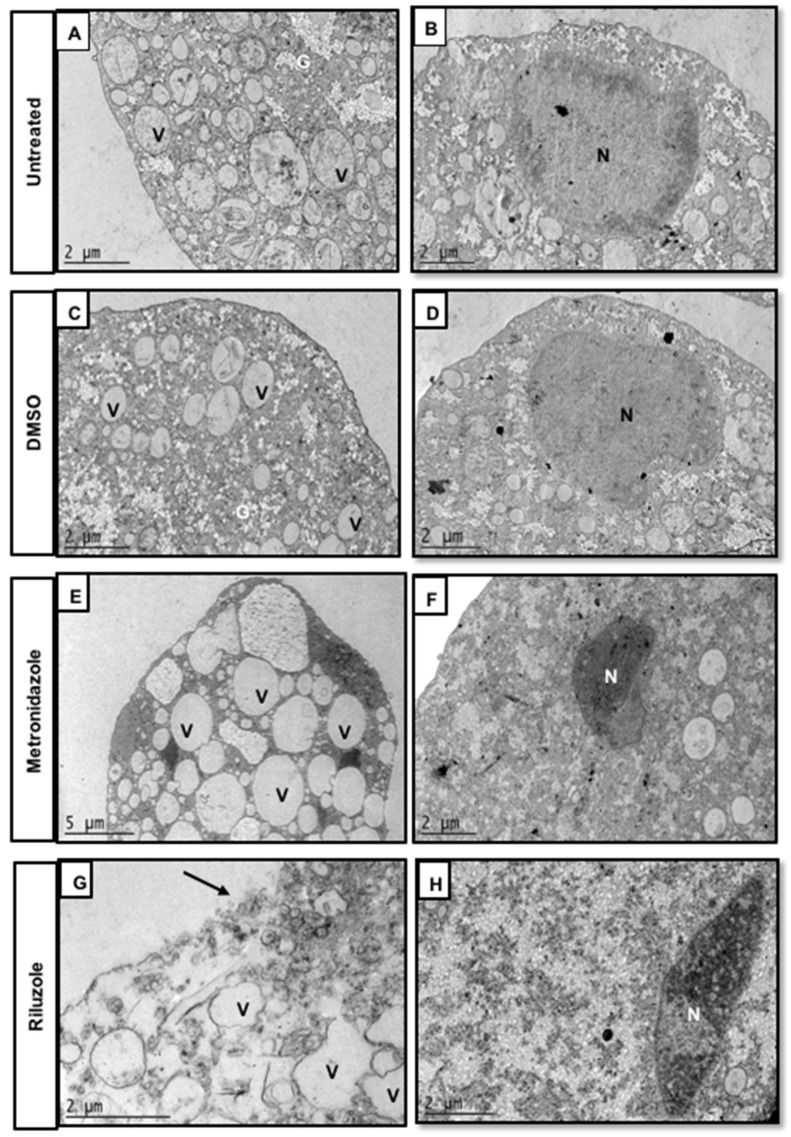
Ultrastructural changes in trophozoites treated with riluzole. Transmission electron microscopy of trophozoites *E. histolytica*. (**A**,**B**) Trophozoite untreated. (**C**,**D**) Trophozoite treated with DMSO. (**E**,**F**) Trophozoite treated with metronidazole IC_50_. (**G**,**H**) Trophozoites were treated with riluzole IC_50_ for 5 h. The amoeba showed lysis (**G**), and the nucleus showed an elongated shape (**H**). The panels showed representative images of the cytoplasm and nucleus. V: vacuole, G: glycogen deposit, N: nucleus, and arrows: the plasma membrane of the amoeba was fragmented.

The ultrastructural changes associated with the anti-amoebic effect of RLZ IC_50_ for 5 h were assessed by transmission electron microscopy (Figure 3). In trophozoites incubated only with fresh medium (untreated) and DMSO, the continuity of the plasma membrane was observed to be well-defined and uniform. In the cytoplasm, there are abundant vacuoles (V) of different sizes and spherical appearance. In addition, in the cytosol, there are scattered glycogen deposits (G) (Figure 3A,C) and a normal-appearing nucleus (N) with its heterochromatin located peripherally (Figure 3B,D), which is characteristic of a typical amoeba nucleus. In contrast, the trophozoites treated with MTZ IC_50_ showed an irregular plasma membrane appearance, an increase in the number of vacuoles and glycogen deposits (Figure 3E), and the nucleus was smaller and its chromatin more electron-dense (Figure 3F), which could indicate that the nucleus was altered and showed signs of pyknosis. The trophozoites treated with RLZ showed morphological alterations, such as a loss of continuity of the plasma membrane (Figure 3G). Additionally, the vacuoles (V) had pleomorphic and diffuse shapes (Figure 3G), and the nucleus (N) had irregular shapes and electron-dense chromatin (Figure 3H), indicating that the nucleus was altered. Additionally, we observed the total lysis of the amoebae, suggesting that all these morphological alterations led to cell death.

### 2.4. Riluzole Treatment Induces Apoptosis in E. histolytica Trophozoites

**Figure 4:**
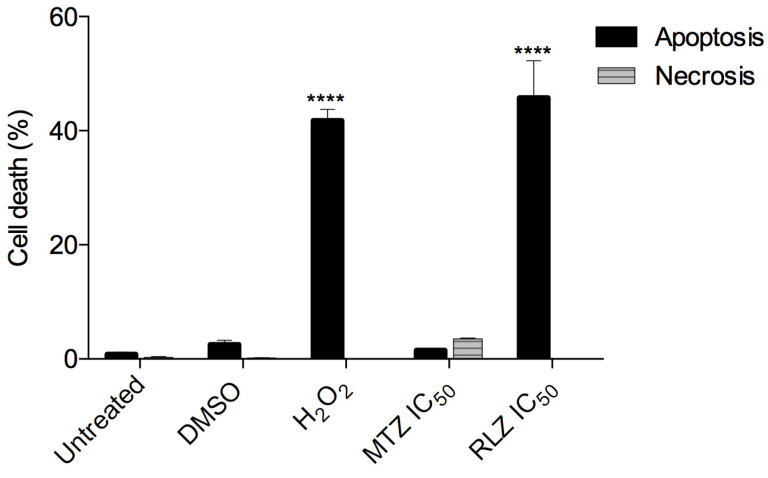
Riluzole induced apoptosis in *E. histolytica* trophozoites. Trophozoites were untreated or treated with the vehicle (0.01% DMSO), as a negative control, MTZ IC_50_ and 2.0 mM hydrogen peroxide (H_2_O_2_) as a positive control, and riluzole IC_50_ for 5 h. The data represent the mean and SD of the percentage of apoptotic and necrotic cells determined by flow cytometry from independent experiments in triplicate. **** *p* < 0.0001. The *p*-values were calculated using 2-wayANOVA with Bonferroni’s test.

We investigated whether the decreased cell viability in trophozoites treated with RLZ was secondary to apoptosis or necrosis. For that purpose, the cells were stained with annexin V as a specific marker for early apoptosis and PI to mark the cells with late apoptosis and necrosis. The data showed that trophozoites untreated or treated with the vehicle (DMSO) displayed 0.83 ± 0.6% and 2.53 ± 1.27% apoptosis, respectively. Riluzole significantly increased the percentage of apoptosis (45.77 ± 11.25%) compared to the negative controls (*p* < 0.0001) after 5 h of incubation (Figure 4), and this result was similar to the trophozoites treated with H_2_O_2_ (positive control), which showed 41.77 ± 3.37% apoptosis (*p* < 0.0001 vs. the control (Figure 4). Unlike riluzole, metronidazole only induced 1.53 ± 0.40% apoptosis and 3.5 ± 0.21% necrosis at the IC_50_ (459.5 µM) for 5 h.

### 2.5. Riluzole Produces ROS and ON in E. histolytica Trophozoites

**Figure 5:**
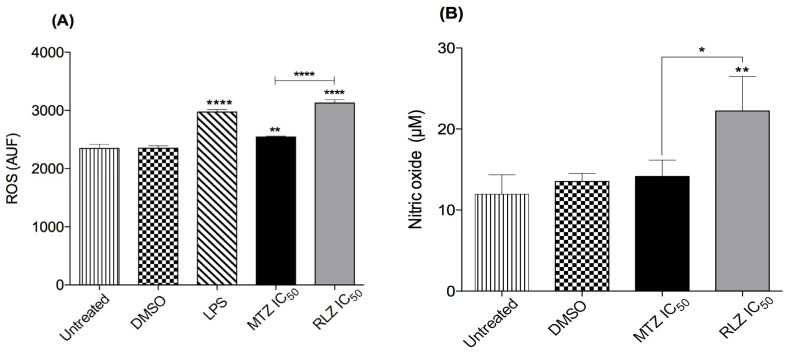
Determination of reactive oxygen species (ROS) and nitric oxide (NO) in *E. histolytica* trophozoites treated with riluzole after 5 h. (**A**) Quantification of ROS in trophozoites untreated and treated with 0.01% dimethyl sulfoxide (DMSO) as the negative controls, metronidazole (MTZ) IC_50_ and 20 µg/mL of lipopolysaccharides (LPS) as the positive controls, or riluzole (RLZ) IC_5o_; data were reported as arbitrary units of fluorescence (AUF). (**B**) Detection of NO in trophozoites untreated and treated with 0.01% DMSO as the negative controls, MTZ IC_50_ as a positive control, or RLZ IC_50._ Values are the means and SD in triplicate of two independent experiments. * *p* < 0.05, ** *p* < 0.01, and **** *p* < 0.0001.

The ROS and NO production were evaluated, because these molecules could be involved in oxidative stress caused by benzothiazole derivatives, which could be an important contributor to its amebicide effect. Accordingly, we measured the ROS level in the supernatants of trophozoites treated with RLZ using a DCF-ROS kit. Basal DCF fluorescence levels were detected in trophozoites in the fresh medium only and were treated with DMSO after 5 h of incubation (Figure 5A). As expected, the positive control with LPS produced a large amount of ROS. Similarly, when trophozoites were treated with RLZ IC_50_, an increment in ROS production was reflected by the increase in their fluorescence. In contrast, trophozoites treated with MTZ IC_50_ considerably reduced ROS production (*p* < 0.0001) (Figure 5A). These results indicate that RLZ induces ROS production in the supernatant of *E. histolytica* trophozoites. To analyze the production of NO in trophozoites treated with RLZ, we used a detection kit to quantify the NO present in the supernatants following the reduction of nitrate to nitrite using the Griess method. Trophozoites with fresh medium showed a basal NO production of 11.96 μM at 5 h, and those treated with DMSO produced 13.51 μM (Figure 5B). Furthermore, when the trophozoites were treated with RLZ IC_50_, the NO production was 22.22 μM, unlike trophozoites treated with MTZ IC_50_, which produced only 14.14 μM nitric oxide. These results demonstrated that trophozoites treated with RLZ can induce the production of NO (Figure 5B).

### 2.6. Riluzole Decreased the Relative Gene Expression of Antioxidant Enzymes

**Figure 6:**
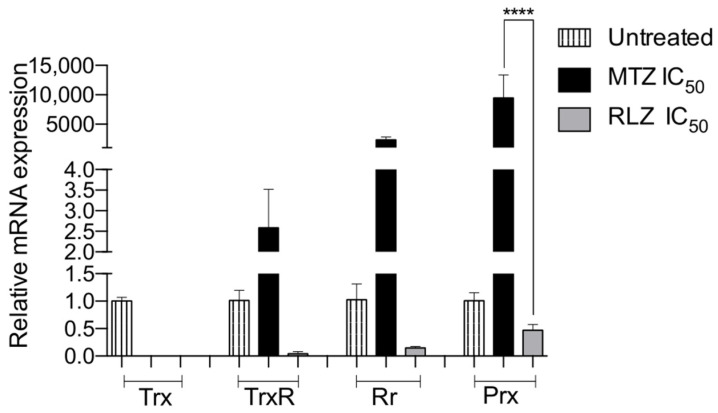
Relative mRNA expression of thioredoxin (Trx), thioredoxin reductase (TrxR), rubrerythrin (Rr), and peroxiredoxin (Prx) in *E. histolytica* treated with riluzole (RLZ) IC_50_ by RT-qPCR. Trophozoites untreated were considered the negative control and trophozoites treated with metronidazole (MTZ) IC_50_ as the positive control. Values represent the mean and standard deviation in triplicate of two independent assays. **** *p* < 0.0001.

To analyze the relative gene expression levels of the antioxidant enzymes in *E. histolytica* trophozoites treated with RLZ compared to the untreated trophozoites, we performed RT-qPCR. Single peaks were observed in the melting curve for each tested gene, indicating a unique PCR product (data not shown). GAPDH was used as a housekeeping gene for data normalization. The relative gene expression of all the antioxidant enzymes decreased following the treatment with RLZ IC_50_. A dramatic decrease of 500-, 25-, 7-, and 2-fold in Trx, TrxR, Rr, and Prx, respectively, for the untreated trophozoites was detected (Figure 6). In contrast, *E. histolytica* trophozoites treated with metronidazole showed an overexpression of 2-, 2000-, and 9000-fold in TrxR, Rr, and Prx, respectively, and only a lightly decreased (1-fold) expression of Trx was detected (Figure 6).

### 2.7. In Silico Molecular Docking Show That Riluzole Has a High Affinity for the Antioxidant Enzymes of E. histolytica

**Figure 7:**
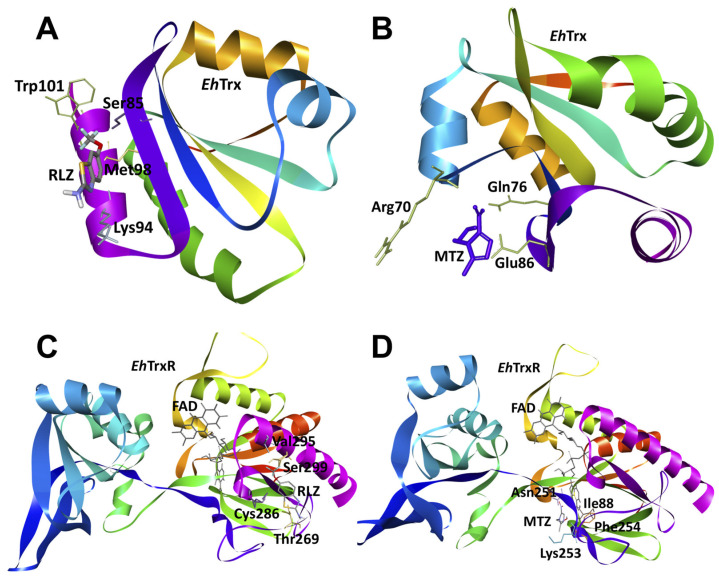
Molecular docking between antioxidant enzymes *Eh*Trx and *Eh*TrxR from *E. histolytica* and RLZ or MTZ. (**A**) Binding mode of the complex between *Eh*Trx and RLZ, binding energy (ΔG) = −5.1 kcal/mol. (**B**) Binding mode of the complex between *Eh*Trx and MTZ, binding energy (ΔG) = −4.2 kcal/mol. (**C**) Binding mode of the complex between *Eh*TrxR and RLZ, binding energy (ΔG) = −6.0 kcal/mol. (**D**) Binding mode of the complex between *Eh*TrxR and MTZ, binding energy (ΔG) = −5.6 kcal/mol. The amino acids (stick format) involved in the interactions with the drugs are labeled in all cases.

To explore the possible interactions between the drugs metronidazole and riluzole with antioxidant enzymes from *E. histolytica*: thioredoxin (*Eh*Trx), thioredoxin reductase (*Eh*TrxR), rubrerythrin (*Eh*Rr), and peroxiredoxin (*Eh*Prx), we carried out an in silico study with molecular docking for each protein studied in this work. The analysis of the interactions showed that riluzole (RLZ) had a higher affinity for the proteins evaluated in all cases compared to metronidazole (MTZ). Particularly for *Eh*Trx, both MTZ and RLZ had an affinity for a region of the protein very similar to each other that, together, were from residues Arg70, Gln76, Ser85, Glu86, Lys94, Met98, and Trp101 (Figure 7A,B); however, this region of the protein was opposite to the reported redox-active site where the cysteines Cys30 and Cys33 were involved in catalytic activity. In the case of *Eh*TrxR, we found a favorable binding mode in terms of energy for RLZ, since, according to our analysis, it binds to residues close to FAD Ser299, Val295, Trh269, and Cys286 on the external face of the protein, while MTZ binds to FAD, Ile88, Asn251, Phe254, and Lys253 but with a lower affinity (Figure 7C,D).

**Figure 8:**
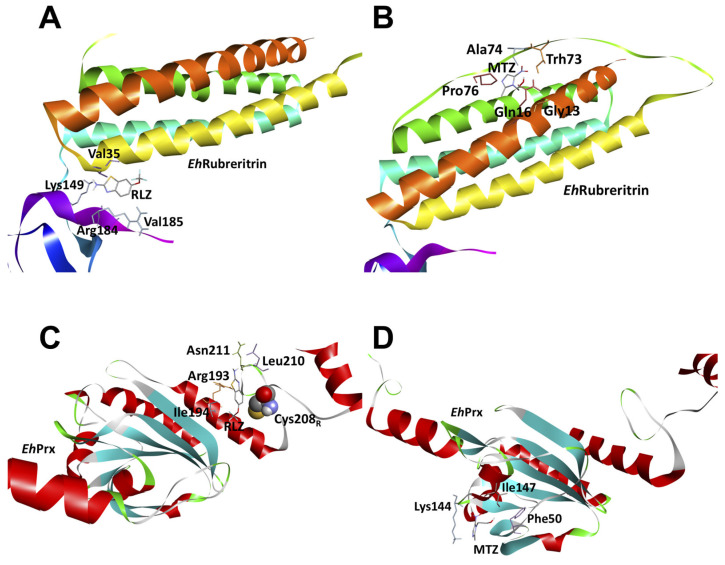
Molecular docking between antioxidant enzymes EhRr and EhPrx from *E. histolytica* and RLZ or MTZ. (**A**) Binding mode of the complex between EhRr and RLZ, binding energy (ΔG) = −5.7 kcal/mol. (**B**) Binding mode of the complex between EhRr and MTZ, binding energy (ΔG) = −4.4 kcal/mol. (**C**) Binding mode of the complex between EhPrx and RLZ, binding energy (ΔG) = −5.5 kcal/mol. (**D**) Binding mode of the complex between EhPrx and MTZ, binding energy (ΔG) = −4.5 kcal/mol. The amino acids (sticks format) involved in the interactions with the drugs are labeled in all cases.

On the other hand, for the *Eh*Rr enzyme, we found that RLZ binds mainly in the rubredoxin-like domain at residues Lys149, Arg184, Val185, and Val35 (Figure 7C), while MTZ binds predominantly near the ferritin-like diiron domain at residues Gly13, Gln16, Thr73, Ala74, and Pro76 but with 12-fold less affinity than RLZ (Figure 8A,B). Finally, the molecular docking analysis with *Eh*Prx showed that RLZ has a 10-fold higher affinity than MTZ at residues Arg193, Ale194, Leu210, Asn211, and Cys208 (Figure 8C,D).

## 3. Discussion

Given these data, we deduced that the anti-amoebic effect of RLZ is primarily due to its privileged structure, because it is an aromatic heterocycle that presents a benzene ring fused to a thiazole ring that contains sulfur (S) and nitrogen (N) atoms. The benzothiazole ring is substituted with a trifluoromethyl group (-CF_3_) at R_1_, a group of (H) on R_2_, and an amino group (CH_3_) at the R_3_ position. Several studies have indicated that fluorine is a highly electronegative, lipophilic halogen atom and has an important effect on the distribution, basicity, or acidity of adjacent groups [37,38]. It also promotes electrostatic interactions due to its affinity for receptors [39] and increases the diffusion velocity across biological membranes [40]. In addition, the amino group (NH_2_) can increase the polarity and solubility in water. The previously described properties give a greater selectivity and synergistic action to the molecule, which can help to increase its anti-amoebic potential.

Our study showed that the viability of *E. histolytica* treated with RLZ decreased significantly, obtaining an IC_50_ = 319.5 μM at 5 h of incubation compared to MTZ (IC_50_ = 459.5 μM), considering that MTZ is a drug of excellence against amoeba. The antiparasitic activity of benzothiazole derivatives have been previously demonstrated; RLZ has an amoebicidal effect on *Acanthamoeba castellanii* cysts and trophozoites [21,22,23]. Moreover, the effect of RLZ has been evaluated on *Leishmania*, and their results show its leishmanicidal effect with an ED_50_ of 25.2 ± 2.6 μg/mL for *L. major* and 23.3 ± 6.9 μg/mL for *L. mexicana* [24]. The compound benzothiazole derivative 2-methoxy-4-[5-(trifluoromethyl)-1,3-benzothiazol-2-yl] phenol (BT10) was evaluated for *Trypanosoma cruzi*. It was found to inhibit the proliferation of epimastigote (IC_50_ (Epi) = 23.1 ± 1.75 μM) and trypomastigote (IC_50_ (Tryp) = 8.5 ± 2.9 μM) [28,29,30]. In another study, the effect of benzothiazole derivatives on *Giardia lamblia* and *Trichomonas vaginalis* was investigated, the benzothiazoles exhibited good giardicidal activity (IC_50_ = 4.627 ± 1.67 μM) and antitrichomonal activity (7.68 ± 3.059 μM) at 48 h of incubation [22]. Furthermore, the anti-plasmodial activity of benzothiazole was shown, and benzothiazole analogs had excellent activity against chloroquine-resistant *P. falciparum* W2 and K1 strains, with IC_50_ values ranging from 7 to 22 nM [41].

However, to exclude the possibility that the RLZ concentrations could affect the cells, we evaluated their cytotoxic effect on the VERO cell line, and no damage was observed, which indicates that RLZ does not cause toxic effects on this cell line. Our results were consistent with those reported by Ozpinar et al. 2021 [23], who mentioned that three benzothiazole-derived compounds did not cause cytotoxic effects on the human fibroblast cell line (WI-38), even at 0.1%. Additionally, its cytotoxic effect has been tested on other cell lines, such as human embryonic fibroblasts of mammals, corroborating that it does not cause damage to the cells [30].

We analyzed the ultrastructural changes caused by RLZ in *E. histolytica* trophozoites. The results showed a rupture of the cytoplasmic membrane, the presence of pleomorphic vacuoles, and damage to the nucleus, ultimately leading to amoebic lysis. DNA damage by benzothiazole derivatives has been previously described by Racané et al. 2021 [42]. In subsequent studies, researchers have found that the crystal structure of the benzothiazole derivative BT6 (2-(4-hydroxy-3-methoxyphenyl)-benzothiazole) adopts a helical arrangement formed by intermolecular O13-H13∙∙N3 hydrogen bonds, suggesting that this compound can intercalate into DNA [43]. They also determined that the benzothiazole derivative BT3 targets the DNA of the kinetoplast of *Trypanosoma cruzi*, inducing damage in 42% of parasites [29]. Our results coincided with other studies in which ultrastructural alterations were evidenced in trophozoites treated with other compounds, such as flavonoids [44,45]. These changes have been previously associated with cell death mechanisms such as apoptosis [46,47]. Currently, there are no previous reports demonstrating that RLZ causes damage leading to trophozoite lysis and, ultimately, cell death. We found that RLZ induces the death of *E. histolytica* trophozoites by apoptosis-like activity, contrary to metronidazole, which induces necrosis. The proapoptotic effect of RLZ has been demonstrated in several types of cancer cells [14,48]. In some works, it has been found that *Giardia intestinalis* trophozoites treated with metronidazole induce apoptosis and necrosis in the same ratio [49], which is concordant with our results. In addition, other studies have reported that different compounds induce cell death by apoptosis in amoebae [50,51]. In contrast to these results, Cuevas et al. 2020 [28] observed that *Trypanosoma cruzi* treated with benzothiazole derivatives did not show signs of cell death by apoptosis, indicating that benzothiazoles act as trypanostatics rather than trypanocides.

ROS and NO production were also evaluated, because it has been described that benzothiazole derivatives could promote oxidative stress in neoplastic liver cells. Thus, hepatocytes could generate ROS that cause damage to trophozoites [52]. Our results indicate that riluzole induces the production of ROS. Recent studies have reported that ROS-induced oxidation of *E. histolytica* actin inhibits its polymerization and leads to a rearrangement of the cytoskeleton [53]; these findings suggest that the increase of ROS could be related to the anti-amoebic effect of RLZ causing ultrastructural changes in the trophozoites described previously. In contrast, Cuevas et al. 2020 [28] reported that compound BT10 does not induce ROS production. In addition, our results showed that incubating *E. histolytica* with RLZ increases the production of NO. Interestingly, this finding was reported by Konstantinova et al. 2018 [54], who indicated that a benzothiazole derivative produced the in vitro release of 69% nitric oxide, showing that this compound is a good candidate for future assays. These findings suggest that the increased production of ROS and NO could be related to the oxidative effect of RLZ, contributing significantly to amoebic damage. *E. histolytica* trophozoites are exposed to an oxidative environment, which is highly toxic to both the parasite and the host cells. Previous studies have indicated that amoebae lack the common antioxidant systems present in eukaryotes, such as catalase, peroxidase, glutathione, and glutathione recycling enzymes [55,56,57], so they rely heavily on the thioredoxin system to combat damage caused by oxidative stress [58]. *E. histolytica* has developed highly efficient enzyme systems to resist oxidative damage and, thus, maintain the intracellular redox balance, with the thioredoxin-dependent system being the best-characterized system in this parasite. This system is involved in biologic processes as protection against oxidative stress, the regulation of DNA synthesis, transcription, cell growth, and programmed cell death [59,60,61]. Thiol-dependent antioxidant enzymes such as Trx, TrxR, Rr, and Prx [58] are involved in redox regulation and are key in the search for new anti-amoebic therapeutic targets such as RLZ. We evaluated the relative gene expression of antioxidant enzymes such as Trx, TrxR, Rr, and Prx in *E. histolytica* trophozoites incubated with RLZ and found, in concordance with the increase in ROS and NO, that the expression of all the antioxidant enzymes decreased, suggesting that they participated in the regulation of the oxidative environment and, at some moments, were depleted, thus decreasing the relative expression of these enzymes and, consequently, their antioxidant capacity to maintain the redox balance in the parasite, ultimately leading to parasite death. Similar to our results, Guerrieri et al. 2013 [24] demonstrated that RLZ has inhibitory activity against the *Leishmania* pteridine reductase enzyme (PTR1), affecting its antioxidant capacity and leading to the death of the parasite. On the other hand, we found that trophozoites treated with MTZ increased the mRNA expression of the antioxidant enzymes Prx, Rr, and TrxR. These results are consistent with a previous report, where it was observed that MTZ increased the gene expression of antioxidant enzymes to protect amoebae from oxidative stress [62]. However, the mRNA expression of those enzymes is not translated into proteins as the result of an activated metronidazole attack on the thiol-based redox system of amoebae [6].

Our molecular docking studies indicated that riluzole could interact with some amino acid residues of the *Eh*Trx, *Eh*TrxR, *Eh*Rr, and *Eh*Prx proteins, finding a high affinity for all the proteins, unlike MTZ. In particular, we highlight the activity of *Eh*Prx, which has 10 times more affinity than MTZ for the amino acid residues Arg193, Ale194, Leu210, Asn211, and Cys208. This cysteine is also called resolving and is found in the catalytic site of the protein in such a way that the binding of RLZ in this region and, particularly, in Cys208 can strongly lead to its inhibition and, consequently, contribute to the accumulation of reactive species, such as has been reported in other works [63]. *Eh*Prx is involved in H_2_0_2_ detoxification [64] by reducing H_2_0_2_ and peroxynitrite [61]. During the detoxification reaction, *Eh*Prx undergoes a peroxide-dependent oxidation, as well as a thiols-dependent reduction cycle. In addition to the highly preserved cysteine, *Eh*Prx possesses a 29 kDa thiol-rich surface antigen. This thiol has been considered as a key element in the protection against H_2_O_2_ [65].

In addition, we found during molecular docking that riluzole had a high affinity for the enzymes *Eh*Trx and *Eh*TrxR. *Eh*TrxR plays an important role in preventing, regulating, and repairing the damage produced by oxidative stress in amoeba [66]. The TrxR enzyme contains a NADPH-binding domain, a FAD domain, and an active site containing a redox-active disulfide [60]. The differences between *Homo sapiens* and *E. histolytica* in the mechanisms of reduction of Trx have allowed the proposal of the system Trx/TrxR as a target molecule for the development of new antiparasitic compounds [67], which is the basis for developing TrxR inhibitors as antiparasitic agents. For example, the drug auranofin (an old drug used for the treatment of human rheumatoid arthritis disease) has been found to demonstrate high efficacy as an anti-amoebic drug, because it inhibits TrxR and blocks Trx reduction, causing increased sensitivity of the parasite to death being mediated by reactive oxygen species [68]. These antioxidant systems have been described as therapeutic drug targets in other parasites, because they can alter the redox balance in a parasite and cause its death [58,69,70]. Furthermore, we found that riluzole binds significantly to *Eh*Rr mainly in the rubredoxin-like domain at residues Lys149, Arg184, Val185, and Val35, reflecting its high affinity, as opposed to MTZ, which is 12-fold lower in affinity. Proteins homologous to Rr possess a rubredoxin-like FeS4 center and a hemerythrin-like binuclear iron cluster with peroxidase activity and participate in H_2_O_2_ detoxification [71]. Previous reports have shown that *E. histolytica* can metabolize oxygen with high affinity without peroxide accumulation [72] or remove hydroperoxides from a cell via cytosolic Prx and the Trx/TrxR system in the absence of catalase and glutathione peroxidase [61,64]. Another mechanism for hydroperoxide detoxification identified in *E. histolytica* is rubrerythrin in mitosomes. Rr is a non-heme iron-containing metalloprotein, which has functional Fe-S centers that allow *Eh*Rr transfer electrons from organic donors to oxygen-reducing hydrogen peroxide. This mechanism prevents the accumulation of hydrogen peroxide and its toxicity in the organelles [71].

Therefore, RLZ could be a promising drug for the treatment of amoebiasis, because it affects the in vitro viability of *E. histolytica* trophozoites through various cellular mechanisms. Moreover, from molecular docking studies, a high affinity to amoeba antioxidant enzymes with RLZ was demonstrated. However, further studies are needed to elucidate its therapeutic target. In addition, it is necessary to evaluate the therapeutic effect of riluzole in an amebiasis-susceptible model, which would support its use as an anti-amoebic drug.

## 4. Materials and Methods

### 4.1. E. histolytica Trophozoite Cultures

The *E. histolytica* trophozoites HMI: IMSS strain was cultured axenically in Diamond’s trypticase yeast iron extract (TYI-S-33) at 37 °C, and the medium was supplemented with 20% heat-inactivated adult bovine serum (S0250, Biowest, Nuaillé, France) and Diamond vitamin tween solution (58980C, Sigma-Aldrich^®^, St. Louis, MO, USA), as previously described [73]. For all the experiments, the trophozoites were harvested during the logarithmic growth phase (72 h) by chilling at 4 °C before use. The trophozoites were concentrated by centrifugation at 300× *g* for 5 min and used immediately. The virulence of the trophozoites was maintained by passing the axenic amoebic cultures through hamster livers twice a month, and the trophozoites were recovered from 7-day-old abscesses and again grown axenically.

#### VERO Cell Culture

The VERO cell line (kidney epithelial cells derived from the African green monkey (*Cercopithecus aethiops*) was obtained from the American Type Culture Collection (ATCC-CRL 1586). The cells were cultivated in Dulbecco‘s modified Eagle’s medium: nutrient mixture F-12 (D-MEM/F-12, 12500-062, Gibco™, Boston, MA, USA) supplemented with 10% fetal bovine serum (FBS) (26140079, Gibco™, Boston, MA, USA) and 1% antibiotic with 10,000 units penicillin and 10 mg streptomycin/mL (P4333, Sigma-Aldrich^®^, St. Louis, MO, USA) at 37 °C in a humidified 5% CO_2_ atmosphere.

### 4.2. Evaluation of the Anti-Amoebic Activity of Riluzole and Its IC_50_ Value

The viability of the amoebae was measured using the water-soluble tetrazolium (WST-1) (Nº cat. 05015944001 Roche Diagnostics, Meylan, France) reagent cell proliferation assay based on the conversion of WST-1 into a water-soluble formazan by the mitochondrial dehydrogenases present in the metabolically active cells. To determine the effect of RLZ on the growth of *E. histolytica*, 3.0 × 10^4^ trophozoites were seeded in 96-well plates and incubated with different concentrations of RLZ (201081C, Santa Cruz, CA, USA) for 5 h at 37 °C. Stock solutions were prepared and dissolved in 0.01% (*v*/*v*) dimethyl sulfoxide (DMSO) (D8418, Sigma-Aldrich^®^, St. Louis, MO, USA) and further diluted with fresh complete medium to set up concentrations of 15 µM, 30 µM, 60 µM, 120 µM, 240 µM, and 480 µM. Trophozoites were incubated with fresh medium only (untreated), treated with 0.01% DMSO (negative control) or MTZ (FLAGYL, Sanofi, México) at concentrations of 15 µM, 30 µM, 60 µM, 120 µM, 240 µM, and 480 µM as the reference drug. After the incubation, the medium was removed, and the trophozoites were washed twice with sterile phosphate-buffered saline (PBS) and 90 μL of PBS pH 6.8, and 10 μL of WST-1 was added to each well plate and incubated for 30 min to 37 °C. The absorbance of the live cells was measured at 450 nm using a microplate reader (BioTek Instruments Synergy HT, Winooski, VT, USA). The absorbance of the background (culture medium + kit reagent) was subtracted from each group. The percentage of viable trophozoites at different concentrations of riluzole was determined by using their optical densities according to the following formula: % viability = [mean Optical Density (O.D.) treated cells × 100]/(mean O.D. control cells). The half-maximal inhibitory concentration (IC_50_) values were calculated by a nonlinear regression analysis (percentage of viability vs. log concentration).

### 4.3. Evaluation of the Cytotoxic Effect of Riluzole on VERO Cells

The RLZ cytotoxicity assays were performed on VERO cells by measuring the mitochondrial dehydrogenase activity in viable cells using the WST-1 technique described above. Cells in the exponential growth phase were detached from the growth surface (trypsin-EDTA, 0.25%, Gibco™ 25200056, Boston, MA, USA) and centrifuged at 750 rpm for 5 min. The cell pellet was resuspended in fresh medium. Cell viability and number were determined by trypan blue staining 0.4% (15250061, Gibco™, Boston, MA, USA) with a hematocytometer for the cell suspension preparation at a density of 10^4^ cells per well for a final volume of 100 μL. This suspension was seeded in 96-well plates (3599, Corning^®^, Corning, NY, USA) and incubated for 24 h before the addition of riluzole. Stock solutions were prepared and dissolved in 0.01% (*v*/*v*) DMSO at the maximum concentration and further diluted with fresh complete medium at the previous concentrations. The cells were treated with different concentrations of RLZ for 5 h at 37 °C in a 5% CO_2_ humidified incubator. The VERO cells were incubated with fresh medium only and treated with 0.01% DMSO or different concentrations of MTZ and were included as controls and reference drugs, respectively. Following the incubation, the medium was removed, and the cells were washed twice with sterile 1X PBS. The cytotoxic effect was determined using a stock solution of WST-1 and incubated for 30 min at 37 °C in a humidified 5% CO_2_ atmosphere. The metabolized WST-1 product was quantified by absorbance measurements from a microplate reader (Bio-Tek Instruments Synergy HT, Winooski, VT, USA) at 450 nm. The percentage viability of VERO cells was determined by inserting the optical densities into the formula described above.

### 4.4. Evaluation of E. histolytica Trophozoites Ultrastructure

To analyze their ultrastructural alterations, the trophozoites (1 × 10^6^ in 0.2 mL of TYI-S-33 medium) were treated with RLZ IC_50_ for 5 h at 37 °C; untreated trophozoites and trophozoites with DMSO and MTZ IC_50_ were included as controls. The trophozoites were washed twice with PBS and fixed with 2.5% glutaraldehyde in 0.1 M cacodylate buffer (pH 7.4). The samples were postfixed for 1 h with 1% (*w*/*v*) osmium tetroxide in cacodylate buffer. The trophozoites were pelleted by centrifugation, dehydrated with ethanol at increasing concentrations and propylene oxide, embedded in Poly/bed^®^ epoxy resin, and polymerized at 60 °C for 24 h. Semithin sections (0.5 µm) were stained with toluidine blue for observation using an optical microscope (Nikon, Eclipse Ci, Tokyo, Japan), and thin sections measuring 60–90 nm were contrasted with uranyl acetate and lead citrate and observed under a Jeol JEM-1011 transmission electron microscope (Jeol, Ltd., Tokyo, Japan).

### 4.5. Determination of the Cell Death Type of E. histolytica Trophozoites

To determine whether RLZ led to amoebic apoptotic/necrotic death, a flow cytometry analysis was performed using the annexin-V-fluorescein isothiocyanate (FITC) and propidium iodide (PI) assays. For that purpose, 1 × 10^6^ trophozoites were cultured in 2 mL of TYI-S-33 medium in 24-well plates and incubated with the medium, the vehicle (0.01% DMSO) as the negative control, 2.0 mM hydrogen peroxide (H_2_O_2_) as the positive control [74], and MTZ IC_5O_ as the reference drug or RLZ IC_50_ for 5 h at 37 °C. For the cytofluorometric analysis, trophozoites were resuspended in 500 μL of 1X × annexin V-binding buffer (Invitrogen™, V13246, Life Technologies, Carlsbad, CA, USA), stained with 5 μL annexin V-FITC (Becton Dickinson Pharmingen™, 556419, San Jose, CA, USA), and incubated for 15 min at room temperature in the dark. Then, 5 μL of propidium iodide (PI) (Becton Dickinson Pharmingen™, 556463, San Jose, CA, USA) was added before beginning the analysis in the cytometer. Lastly, the samples were acquired using a FACSAria Fusion cytometer (Beckton Dickinson) and analyzed using BD FACSDiva 8.0.2 software (BD Biosciences, Heidelberg, Germany). Annexin V–FITC-stained trophozoites were detected by flow cytometry (excitation wavelength, 488 nm; emission wavelength, 520 nm) using a FITC signal detector (FL1), and PI staining was detected by a phycoerythrin emission signal detector (FL2). At least 1 × 10^4^ gated events in the dot plot of FSC-SSC were analyzed, and the data were reported as the percentage of single-positive cells for Annexin V and double-positive cells for Annexin V/PI. Single-positive cells for PI were computed until necrosis. This assay was recorded in triplicate, and the results corresponded to three independent experiments.

### 4.6. Determination of Reactive Oxygen Species (ROS)

The ROS production was determined by employing a 2′, 7′-dichlorodihydrofluorescein (DCF) ROS assay kit (Ab238535, Abcam, Cambridge, UK). ROS production was quantified in trophozoites cultured in 3 × 10^4^ cells of fresh D-MEM/F-12 medium in 24-well culture plates and treated with the concentration corresponding to the RLZ IC_50_ for 5 h at 37 °C as described above. Trophozoites co-incubated with 20 µg/mL of lipopolysaccharide (LPS) were used as a positive control for ROS production (L3129, Sigma-Aldrich^®^, St. Louis, MO, USA). For the controls, we used supernatants from *E. histolytica* trophozoites incubated with fresh medium only and incubated with 0.01% DMSO as the vehicle and MTZ IC_5O_ as the reference drug. In brief, 50 μL of supernatant and 50 μL of the catalyst solution kit were added to 96-well black plates, mixed well, and incubated for 5 min at room temperature, and 100 μL of DCFH solution (DCF-DiOxyQ and priming reagent) was added before they were kept in the dark for 30 min at 37 °C. The fluorescence intensity was measured on black plates with a spectrofluorometer (BioTek Instruments Synergy HT, Winooski, VT, USA) using 485 nm excitation and 528 nm emission filters.

### 4.7. Determination of Nitric Oxide (NO)

The NO production was determined using a NO assay kit (Ab272517, Abcam, Cambridge, UK) according to the manufacturer’s instructions. In brief, trophozoites were cultured in 3 × 10^4^ cells of fresh TYI-S-33 medium in 24-well culture plates. The amoebae were treated with the concentration corresponding to the RLZ IC_50_ for 5 h at 37 °C. For the controls, we used supernatants from *E. histolytica* trophozoites incubated with fresh medium only and incubated with 0.01% DMSO. Trophozoites were incubated with 20 µg/mL of LPS or MTZ IC_5O_ as the reference drug, and a deproteinization process was performed by adding 150 μL of the sample to a tube, mixing with 8 µL of zinc sulfate (ZnSO_4_), and then adding 8 µL of sodium hydroxide (NaOH) and centrifuging for 10 min. Then, 100 µL of the supernatant was transferred to a new tube, and 200 µL of the working solution was added and incubated for 60 min at 37 °C. In brief, the tubes were centrifuged, and the contents were transferred to 96-well plates. The absorbance was read with a spectrophotometer (BioTek Instruments Synergy HT, Winooski, VT, USA) at 540 nm.

### 4.8. Relative Gene Expression of E. histolytica Antioxidant Enzymes

We evaluated the gene expression of the antioxidant enzymes present in *E. histolytica* from three independent biological replicates incubated with RLZ by RT-qPCR; thus, we searched for putative genetic candidates in the *E. histolytica* genome. The sequences identified here were thioredoxin (Trx) (XM_649815), rubrerythrin (Rr) (XM_647039.2), thioredoxin reductase (TrxR) (XM_650656.2), peroxiredoxin (Prx) (XM_644379.2), and the gene glyceraldehyde-3-phosphate dehydrogenase (GAPDH AB002800.1), which was used as an endogenous gene. *E. histolytica* (1.5 × 10^6^ cell/mL) were incubated for 5 h with RLZ IC_50_ or MTZ IC_50_, and untreated *E. histolytica* trophozoites were used as the control to extract the total RNA using the TRIzol reagent (Invitrogen™, 15596026, Life Technologies, Carlsbad, CA, USA) according to the manufacturer’s protocol. The RNA concentration was spectrophotometrically determined using a NanoDrop2000 (Thermo Scientific™, Waltham, MA, USA). The isolated RNA was treated with RQ1 RNase-free DNase (M6101, PROMEGA, Madison, WI, USA) to avoid genomic DNA contamination, and cDNA was synthesized using the First Strand cDNA synthesis kit (Thermo Scientific™ K1622, Waltham, MA, USA) according to the manufacturer’s protocol. RT-qPCR was performed with the Step One Real-Time PCR system (Applied Biosystems, Foster City, CA, USA) by monitoring the increase in fluorescence in real time using SYBR Green PCR Master Mix (4309155, Applied Biosystems, Foster City, CA, USA). Melting curve protocols were performed to ensure the specificity of the amplification products. The primers were designed using Primer Express 3.0.1 software (Applied Biosystems, Foster City, CA, USA) and synthesized commercially (IDT Integrated DNA Technologies, Coralville, IA, USA) (Table 1). The relative quantification of the antioxidant enzymes was calculated using the CT method by applying the comparative cycle threshold (CT), which uses the arithmetic formula 2−ΔΔCT [75]. To validate the method, we verified that the amplification efficiency for the target genes and the endogenous gene GAPDH were nearly equal. The statistical significance between the untreated and treated trophozoites was calculated using Bonferroni’s test with GraphPad Prism statistical software version 8.0.2 (GraphPad, San Diego, CA, USA).

### 4.9. Molecular Docking Simulation

To explore the possible interactions between the drugs metronidazole (MTZ) and riluzole (RLZ) with antioxidant enzymes from *E. histolytica*, we performed an in silico molecular docking study using AutoDock Vina (ADV) 1.1.2 [76]. The receptors used for this analysis were thioredoxin (Trx), thioredoxin reductase (TrxR), peroxiredoxin (Prx), and rubrerythrin (Rr). The structures of Trx, Prx, and Rr were taken from the AlphaFold Protein Structure Database (https://alphafold.ebi.ac.uk, accessed on 9 March 2023) with ID entries C4MBD8_ENTHI, CR29_ENTHI, and C4M030_ENTHI, respectively, while TrxR was taken from the RCSB Protein Data Bank (RCSB PDB, https://www.rcsb.org, accessed on 10 March 2023) with PDB ID 4CCQ. The receptors and ligands were prepared and converted to *. PDBQT files in AutoDockTools 1.5.6 [77]. Protein preparation was performed using the standard protocol consisting of the removal of co-crystallized ligands and water molecules (where applicable); polar hydrogens were added, and the Kollman charges for all the receptor atoms were computed to assess the hydrogen-bonding interactions. All the other parameters were kept at their default settings. The MTZ and RLZ ligands were coupled to each receptor using a grid box and grid center, as specified: for Trx, centered on the midpoint of the protein using grid box 90 × 90 × 90 Å; for TrxR, grid box 50 × 100 × 60 Å centered at X = 4.5336 Å, Y = −16.357 Å, and Z = 1.8136 Å; for Prx centered at the midpoint between the Cα of the peroxidic and resolutive cysteines at X = −7.7225 Å, Y = 5.1735 Å, and Z = 2.1775 Å using grid box 100 × 100 × 80 Å; and for Rr centered on the midpoint of the protein using grid box 126 × 126 × 126 Å. The ADV algorithm was used to find the best complex between ligands and proteins. A maximum of 20 conformers was considered for each ligand, and the complex with the lowest free binding energy was selected to analyze the interactions and modes of binding between antioxidant enzymes from *E. histolytica* and MTZ or RLZ using MGLTools 1.5.6 and Discovery Studio viewer v21.

### 4.10. Statistical Analysis

The statistical analysis was performed with GraphPad Prism statistical software version 8.0.2 (GraphPad, San Diego, CA, USA). The data were expressed as the means and standard error (SD) for a minimum of two independent experiments, each in technical triplicate. In the comparisons between more than two groups, we used one-way analysis of variance (ANOVA) and Bonferroni’s test. Statistical significance was defined as * *p* < 0.05, ** *p* < 0.01, *** *p* < 0.001, and **** *p* < 0.0001.

## Figures and Tables

**Table 1:** Primers sequences for the antioxidant enzymes using the RT-qPCR assay.

Gene	Accession	Forward (5′ → 3′)	Reverse (5′ → 3′)
Thioredoxin	XM_649815.1	TAT GC AGA GTG GTG TGG TCC AT	AAA TGT CGG CAT ACA ACG AAT ACC
Rubrerythrin	XM_647039.2	ATG CTC AAA TTG CTG CTA GAC TT	ATA TCC ACA TTC TCT ACA AAC CCA AA
Thioredoxin reductase	XM_650656.2	ATG AGA ACA CAA TCA GAG AAG TAT GGA	AGC TGT AGC ACC TGT TGC AAT AAT
Peroxiredoxin	XM_646911.2 XM_644418.2 XM_643430.2	CGA AGC AGG AAT TGC AAG AAG	GCT CCA TGT TCA TCA CTG AAT TG
GAPDH	AB002800.1	TTC ATG GAT CCA AAA TAC ATG GTT	GCC AAT TTG AGC TGG ATC TCT T

## Data Availability

The data are contained within the article.
